# Th Cells Promote CTL Survival and Memory via Acquired pMHC-I and Endogenous IL-2 and CD40L Signaling and by Modulating Apoptosis-Controlling Pathways

**DOI:** 10.1371/journal.pone.0064787

**Published:** 2013-06-13

**Authors:** Channakeshava Sokke Umeshappa, Yufeng Xie, Shulin Xu, Roopa Hebbandi Nanjundappa, Andrew Freywald, Yulin Deng, Hong Ma, Jim Xiang

**Affiliations:** 1 Cancer Research Unit, Saskatchewan Cancer Agency, Saskatoon, Saskatchewan, Canada; 2 Department of Pathology, University of Saskatchewan, Saskatoon, Saskatchewan, Canada; 3 School of Life Science, Beijing Institute of Technology, Beijing, China; J. Heyrovsky Institute of Physical Chemistry, Czech Republic

## Abstract

Involvement of CD4^+^ helper T (Th) cells is crucial for CD8^+^ cytotoxic T lymphocyte (CTL)-mediated immunity. However, CD4^+^ Th’s signals that govern CTL survival and functional memory are still not completely understood. In this study, we assessed the role of CD4^+^ Th cells with acquired antigen-presenting machineries in determining CTL fates. We utilized an adoptive co-transfer into CD4^+^ T cell-sufficient or -deficient mice of OTI CTLs and OTII Th cells or Th cells with various gene deficiencies pre-stimulated *in vitro* by ovalbumin (OVA)-pulsed dendritic cell (DCova). CTL survival was kinetically assessed in these mice using FITC-anti-CD8 and PE-H-2K^b^/OVA_257-264_ tetramer staining by flow cytometry. We show that by acting via endogenous CD40L and IL-2, and acquired peptide-MHC-I (pMHC-I) complex signaling, CD4^+^ Th cells enhance survival of transferred effector CTLs and their differentiation into the functional memory CTLs capable of protecting against highly-metastasizing tumor challenge. Moreover, RT-PCR, flow cytometry and Western blot analysis demonstrate that increased survival of CD4^+^ Th cell-helped CTLs is matched with enhanced Akt1/NF-κB activation, down-regulation of TRAIL, and altered expression profiles with up-regulation of prosurvival (Bcl-2) and down-regulation of proapoptotic (Bcl-10, Casp-3, Casp-4, Casp-7) molecules. Taken together, our results reveal a previously unexplored mechanistic role for CD4^+^ Th cells in programming CTL survival and memory recall responses. This knowledge could also aid in the development of efficient adoptive CTL cancer therapy.

## Introduction

CD8^+^ T cells play a defensive role against infectious and cancer diseases. Following recognition of foreign antigen (Ag), they undergo 3 distinct phases of immune responses [1,2]: (i) a proliferation (priming) phase in which naïve CD8^+^ T cells undergo autonomous clonal expansion and develop into effector cytotoxic T lymphocytes (CTLs); (ii) a contraction phase, in which ~95% of effector CTLs undergo activation-induced cell death (AICD) through apoptosis, allowing development of ~5-10% memory CTLs; and (iii) a maintenance (memory development) phase in which memory CTLs survive for a prolonged duration. In contrast to their naïve counterparts, memory CTLs respond swiftly by rapid proliferation and heightened effector functions in recall responses to subsequent Ag encounters.

CD4^+^ T cells have potential to influence multiple aspects of CTL responses. Their importance in primary CTL responses was initially demonstrated in immunizations with non-inflammatory Ags such as male minor-HY and Qa-1 alloantigen [3]. The requirement for cognate CD4^+^ T cell help in different phases of CTL responses is frequently debated and appears to vary, depending on the immunization types. In the absence of inflammation, antigen-presenting cells (APCs) have to be activated by CD4^+^ T cells through CD40/CD40L interactions to prime CD8^+^ CTL responses [4,5]. Alternatively, cognate CD4^+^ T cells have also been shown to initiate a direct signaling in CD40-expressing CD8^+^ T cells through CD40L costimulation [6–8]. Although CD4^+^ T cell help can be dispensable for primary CTL generation, it is prerequisite for programming memory CTLs in most situations [2,6,9–11]. As the effector phase constitutes both AICD and memory CTL development, APC-stimulated Th cells appear to play a critical role in effector CTL survival and functional memory development [2,12,13]. Recently, CD4^+^ T cell-provided help was shown to support effector CTL survival through the regulation of the TRAIL and Bcl-x_L_ molecules [2,9,14]. However, the exact molecular mechanism of CD4^+^ T cell help that modulates effector CTL survival is still not completely understood.

Intercellular membrane transfer through the process of trogocytosis is a wide-spread phenomenon in the immune system and plays a crucial role in immunomodulation [15–18]. Recently, acquisition of the Ag-presenting machinery (APM) of APCs by CD4^+^ T cells has attracted a strong attention [1,8,16,19–25], and the functional role of acquired APM is being actively investigated. Although not being efficient in soluble-Ag capture, CD4^+^ T cells acquire APM from APCs, and present Ags to other naïve CD4^+^ T cells, inducing their activation and proliferation [8,17,25,26]. In contrast, Ag presentation to previously Ag-experienced, activated or memory CD4^+^ T cells inhibits proliferation, thereby maintaining the homeostasis of immune responses [1,8,19,23]. In our previous reports, we demonstrated the role for CD4^+^ T cell-acquired APM in modulating responses of naïve CD8^+^ T cells [8,21,27]. We showed that APC-stimulated CD4^+^ helper T (Th) cells are able to stimulate naïve CD8^+^ T cells via acquired peptide-major histocompatibility complex-I (pMHC-I) complexes, inducing central memory CTL stimulation and anti-tumor immunity. However, the impact of this acquired APM on the behavior of previously Ag-experienced effector CTLs is not well understood, while this information could be crucial for the development of the successful adoptive CTL cancer therapy.

Here, we generated effector Th cells and CTLs by *in vitro* cultivation of transgenic OTII CD4^+^ and OTI CD8^+^ T cells with ovalbumin (OVA)-pulsed dendritic cells (DCova), and investigated whether Th cells with acquired APM have any beneficial effects on effector CTLs after adoptive co-transferring into CD4^+^ T cell-sufficient [wild-type (WT) C57BL/6] or CD4^+^ T cell-deficient [Ia^b-/-^, knockout] mice. We showed that Th cells enhanced CTL survival and their differentiation into functional memory pool, leading to CTL-mediated immune protection against highly metastasizing tumor challenge. In the effector/contraction phase, the inherent ability of CD4^+^ Th cells to provide IL-2 and CD40L and the low level of acquired pMHC-I complexes was found to be critical for endowing CTLs with survival, and memory development advantages. The down-stream molecular mechanisms underlying the prolonged survival of Th-helped CTLs were also systematically assessed.

## Materials and Methods

### Ethics statement

All the animal experiments were performed as per the guidelines approved by the University Committee on Animal Care and Supply, University of Saskatchewan. Protocol Number: 20100027.

### Reagents, tumor cells and animals

The biotin- and fluorescent dye-labeled (FITC or PE) antibodies (Abs) specific for CD4 (GK1.5), CD11c (HL3), CD44 (IM7), H-2K^b^ (AF6-88.5), Ia^b^ (KH74), CD80 (16-10A1), CD40 (3/23), CD40L (MR1), CD54 (3E2), CD62L (MEL-14), CD69 (H1.2F3), TRAIL (N2B2), PD-1 (J43) and IFN-γ (XMG1.2), and streptavidin-PE-Texas-Red, PE-Cy5 or -FITC were purchased from BD-Biosciences or eBiolegend. The biotin-anti-IL-7Rα (A7R34) and -peptide/MHC-I (pMHC-I) (25-D1.16) Abs were purchased from eBioscience. The FITC-anti-perforin (CB5.4) Ab was obtained from Alexis Chemicals. The FITC-anti-CD8 (KT15) Ab and H-2K^b^/OVA_257-264_ tetramer were obtained from Beckman Coulter. The OVA-transfected, mouse malignant melanoma (BL6-10_OVA_) and EL4 (EG7) cell lines were cultured as described previously [8]. The mouse B-cell-hybridoma cell line LB27 expressing both H-2K^b^ and Ia^b^ was obtained from American Type Culture Collection. The age-matched wild-type (WT) C57BL/6 (B6, CD45.2^+^), OVA_257-264_ and OVA_323-329_-specific TCR-transgenic OTI and OTII, H-2K^b-/-^ and Ia^b-/-^ mice on WT B6 background were purchased from Jackson Laboratory. The OTII/CD40L^-/-^ and OTII/IL-2^-/-^ mice were generated by backcrossing designated gene-deficient mice with OTII mice [8].

### Preparation and characterization of mature DCova

Bone-marrow-derived, DCova from WT mice were generated by culturing bone marrow cells for 6 days in medium containing IL-4 (20 ng/ml) and GM-CSF (20 ng/ml) and pulsing with 0.1 mg/mL OVA overnight at 37°C as described previously [21]. OVA-pulsed DCs generated from WT B6 and H-2K^b-/-^ mice were referred as DCova and DCova(K^b-/-^), respectively.

### Preparation of naïve and effector CD4^+^ or CD8^+^ T cells

The naïve CD4^+^ and CD8^+^ T cells were isolated from WT, OTII or OTI splenocytes as previously described [8]. To generate active, OVA-specific CD8^+^ T (effector CTL) and Th cells, the OTI CD8^+^ or OTII CD4^+^ T cells (0.75X10^6^ cells/mL, 200 µl/well) were respectively cultured with irradiated (4,000 rads) DCova (0.75X10^6^ cells/mL, 50 µl/well) at 1:4 ratio for three days as previously described [21]. The activated CD4^+^ and CD8^+^ T cells were purified by Ficoll-Paque (Sigma-Aldrich) separation followed by using CD4 and CD8 MACS microbeads (Beckman Coulter) and termed effector CD4^+^ Th cells and CTLs, respectively. The naïve, effector CTL and Th cells were stained with panel of cell-, naïve-, activation- or memory-specific markers, and characterized phenotypically by flow cytometry. The Th cells derived from OTII/CD40L^-/-^ and OTII/IL-2^-/-^ mice were termed Th(CD40L^-/-^) and Th(IL-2^-/-^), respectively. DCova(K^b-/-^)-stimulated Th cells without acquired pMHC-I molecules were referred as Th(pMHC-I^-/-^). For cytokine profiling, effector CTL and Th or Th(IL-2^-/-^) were respectively re-stimulated with irradiated EG7 and OVAII (OVA_323–339_, Multiple Peptide Systems)-pulsed LB27, and culture supernatants were assessed using cytokine ELISA kits (R&D Systems) from previous description with few modifications [28]. Naive OTI CD8^+^ T cells were co-cultured with irradiated DCova in the presence or absence of naïve OTII CD4^+^ T cells for 3 days. For priming studies, the activated CD8^+^ T cells were purified by Ficoll-Paque separation and negative selection using anti-CD4 (L3T4) paramagnetic beads (Dynal), and termed effector CTLs and CTLs with help in *in vitro* priming, respectively.

### In vivo CD8^+^ CTL survival and recall studies

Approximately 5x10^6^ effector CTLs alone that received help during priming period or 5x10^6^ effectors CTLs with or without 2x10^6^ Th cells (i.e., help after CTL priming) were i.v. transferred WT mice. Similarly, effector CTLs alone that received help during priming period or effectors CTLs with or without effector Th, Th(IL-2^-/-^), Th(CD40L^-/-^), and Th (pMHC-I^-/-^) cells (2x10^6^each) were i.v. transferred to Ia^b-/-^ mice. After confirming equal engraftment on the following day, the effector CTL survival was monitored 6 or 60 days later in peripheral blood by staining with H-2K^b^/OVA_257-264_ tetramer and FITC-anti-CD8 Ab (tetramer assay) [8]. In recall studies, all the groups were boosted i.v. with 1x10^6^ DCova 60 days later and monitored for the expansion of memory CTLs on 4^th^ day by tetramer assay. To phenotypically characterize effector or memory CTLs, the blood samples were collected 6 or 60 days later, and stained for tetramer assay along with panel of biotin-conjugated Abs specific for effector or memory markers, and streptavidin-PE-Texas Red.

### Tumor protection studies

To assess functional effect of memory CTLs, approximately 5x10^6^ effector CTLs alone that received help during priming period or 5x10^6^ effectors CTLs with or without 2x10^6^ Th cells (i.e., help after CTL priming) were i.v. transferred WT and Ia^b-/-^ mice. In addition, to understand mechanism of CD4^+^ T cells, some Ia^b-/-^ mice groups were also transferred with effector CTLs with effector Th, Th(IL-2^-/-^), Th(CD40L^-/-^) or Th(pMHC-I^-/-^) cells (2x10^6^each). Hundred days later, all the groups were i.v. challenged with highly-metastasizing BL6-10_OVA_ tumor cells (0.5x10^6^/mice), monitored for protection as described previously, and sacrificed later to determine the numbers of surface black tumor colonies in the lungs [8].

### Intracellular IFN-γ staining

On the 24^th^ day of tumor challenge, the splenocytes of helped or unhelped mice were re-stimulated with OVAI (OVA_257-264_, Multiple Peptide Systems) and subjected to intracellular IFN-γ staining (BD-Biosciences) as described previously [27].

### Purification of unhelped or transitionally helped CTLs

The WT and Ia^b-/-^ mice were i.v. injected with effector CTLs (5x10^6^) with or without effector CD4^+^ Th (2x10^6^) cells. The blood and lymphoid organs were collected 16 days later, and processed to remove RBCs. The T lymphocytes were enriched in nylon wool columns (C & A Scientific). After labeling T lymphocytes with H-2K^b^/OVA_257-264_ tetramer and anti-PE microbeads (Miltenyi Biotec Inc), a highly purified population of OVA-specific CTLs was obtained by positive selection by passing in 2 separate MACS columns sequentially. The purified CTLs from WT or Ia^b-/-^ mice, which were transferred with CTLs plus effector CD4^+^ Th cells were termed “helped CTLs” in WT or Ia^b-/-^ mice, whereas the purified CTLs from Ia^b-/-^ mice transferred with only CTLs were termed “unhelped CTLs” in Ia^b-/-^ mice.

### RT^2^ profiler PCR array system

The expression of pathway-focused panel of 84 genes related to apoptosis in the above three groups of CTLs (helped CTLs in WT or Ia^b-/-^ and unhelped CTLs in Ia^b-/-^) was examined using RT^2^ *Profiler* ^TM^ PCR array (SuperArray Bioscience). Total RNA was isolated using RNeasy extraction kit (Qiagen) and reverse transcribed using RT^2^ First Strand Kit (SuperArray Bioscience). The mRNA expression of each gene in array system was performed using StepOnePlus thermocycler (Applied Biosystems) and analyzed using *Hprt1, Gapdh*, and *β-actin* as internal controls in web-based software as per manufacturer’s instructions.

### qRT-PCR analysis

The cDNA samples of the above three groups of CTLs (helped CTLs in WT or Ia^b-/-^ and unhelped CTLs in Ia^b-/-^) were further subjected to quantitative RT-PCR (qRT-PCR) for validation of the array results. Sequence-specific primers for *β-actin, Bcl-2*, *FasL and TRAIL* were previously described [9,29], and for *TRAIL receptor, Nfκb1, Bcl10, Akt1, Caspase-4* and *-7* were given in supplementary information (Table **S1**). qRT-PCR was performed using SYBR Green method following manufacturer’s protocol. Briefly, 20 ng of cDNA, 50nM of each primer and 1X Master Mix (Applied Biosystems) were used in 25 µl volume. The mRNA expressions were analyzed as described for PCR array using *β-actin* control*.*

### Western blotting

Western blotting was performed in CTLs and unhelped CTLs as described previously with few modifications. The blots were stained with panel of monoclonal- or polyclonal-rabbit Abs specific for Bcl-2 (50E3), β-actin (13E5), Bcl10 (C79F1), Akt1 (2H10), phospho-Akt1 (S473), cleaved Caspase-3 (Asp175) and -7 (Asp198) (Cell Signaling Technology), NF-κB-p65 (C-20), phospho-NF-κB-p65 (Ser536) (Santa Cruz Biotechnology) and NFATc1 (7A6) (BD Biosciences). The blots were incubated with goat anti-rabbit IRDyeR800/680CW, and band densities were quantified using ODYSSEY densitometer (LI-COR Bioscience).

### Statistical analysis

The statistical analysis was performed using Graphpad Prism-3.0. The results are presented as mean ± SD. The statistical significance between two or more groups was analyzed by Student’s *t*-test or analysis of variance, respectively. *p*<0.05 was considered statistically significant.

## Results

In line with previous observations [8], OVA-pulsed bone marrow-derived DCs (DCova), generated in our experiments, expressed all the maturation markers, including Ia^b^, CD40 and CD80 and OVA-specific pMHC-I (Figure **S1a**). Following *in vitro* stimulation of OTII CD4^+^ or OTI CD8^+^ T cells with irradiated DCova, the active OVA-specific CD4^+^ or CD8^+^ T cells were purified using CD4 or CD8 MACS microbeads subsequent to Ficoll separation. The purified Th or effector CTLs had negligible DC contamination [8,30] (Figure **S1b**). DCova-stimulated CD8^+^ T cells displayed their subset marker (CD8), activation marker (CD69) and effector molecule (perforin), but not IL-7Rα or CD62L [30] (Figure **S1a**). In addition, they secreted IFN-γ (3.55±0.61ng/mL/10^6^cells/24hrs) and TNF-α (1.15±0.13ng/mL/10^6^cells/24hrs), but not IL-4 or IL-10 cytokine, indicating a functional effector CTL phenotype. DCova-stimulated CD4^+^ T cells displayed their subset marker (CD4) and activation marker (CD69) and a low level of acquired pMHC-I. These cells secreted IFN-γ (2.1±0.35ng/mL/10^6^cells/24hrs) and IL-2 (2.75±0.47ng/mL/10^6^cells/24hrs), but not IL-4 and IL-10 cytokine, confirming a type 1 T helper (Th1) cell phenotype [8] (Figure **S1a**).

**Figure 1:**
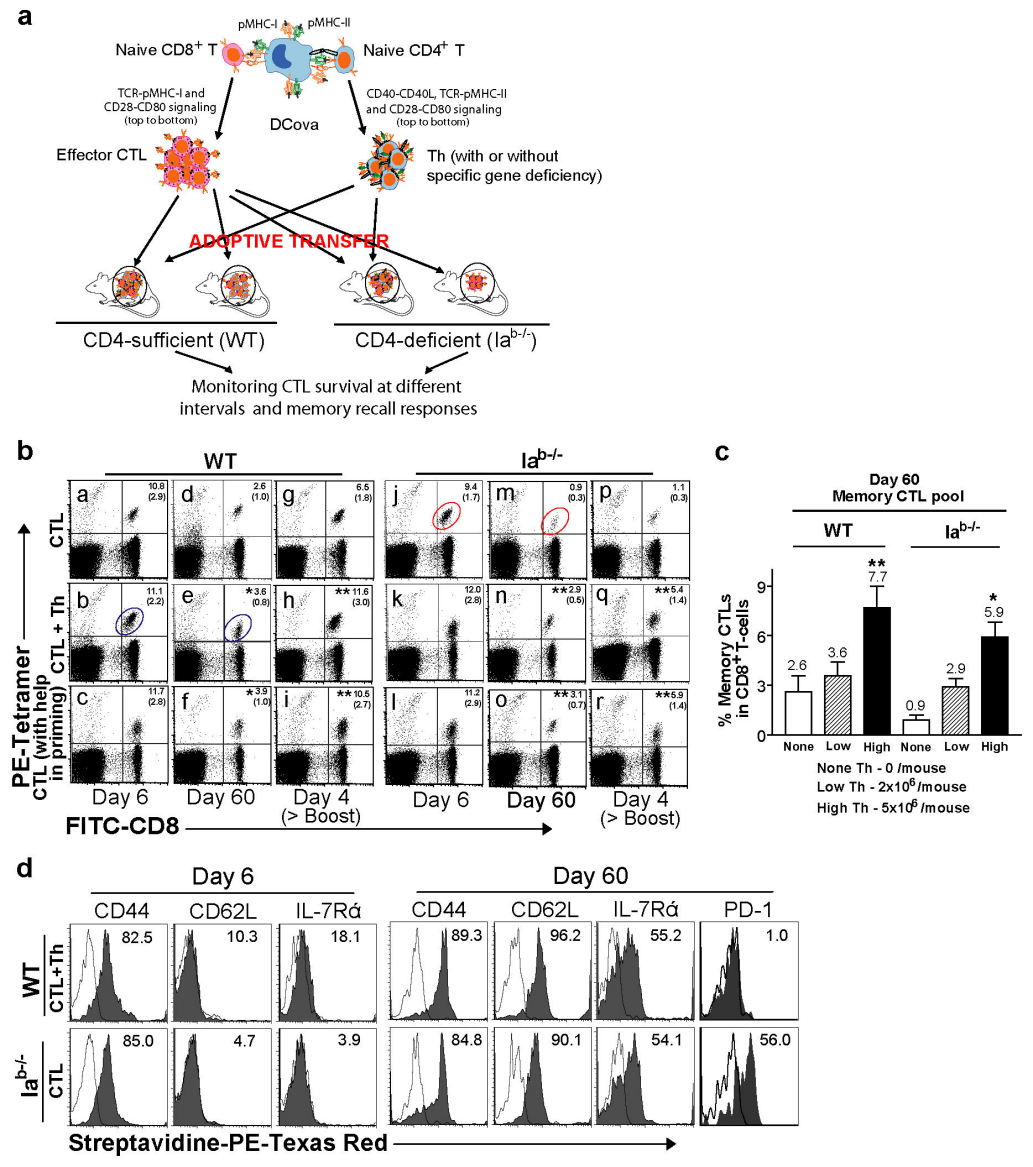
Th cell help provided during the priming or transitional period enhances effector CTL survival and transition to memory development. (**a**) Experimental Design. To determine direct impact of Th cells on effector CTL fates after priming, effector CTLs and Th cells with or without specific gene deficiency were adoptively transferred to WT or CD4^+^ T cell-deficient mice. All mice were monitored for CTL survival and memory CTL recall responses during the memory phase. Memory recall responses were assessed by boosting with DCova 60 days following the adoptive transfer or by challenging with highly metastasizing BL6-10_OVA_ 100 days post-transfer. (**b**) Approximately 5x10^6^ effector CTLs alone that received help during the priming period or 5x10^6^ effectors CTLs without or with 2x10^6^ Th cells (i.e. help after CTL priming) were i.v. transferred into WT or Ia^b-/-^ mice as indicated. Six and sixty days later, the transferred CTLs were monitored in the tetramer assay. On the 60^th^ day, all the groups were boosted with 1x10^6^ DCova and monitored for memory CTL expansion 4 days later. The values represent mean%±(SD) of tetramer^+^ (Ova-specific) CTLs in total CD8^+^ T cell population, and are cumulative of three independent experiments with three to five mice per group. * or **, *p* < 0.05 or 0.01, respectively, versus unhelped CTLs. (**c**) Influence of Th cell numbers on effector CTL survival and memory CTL development. Approximately 5x10^6^ effector CTLs alone (none) or 5x10^6^ effector CTLs together with 2x10^6^ (low) or 5x10^6^ (high) Th cells were adoptively transferred to WT or CD4^+^ T cell-deficient mice and assessed 60 days later in the peripheral blood by tetramer assay. The values represent mean%±SD of tetramer^+^ CTLs in total CD8^+^ T cell population, and are cumulative of two independent experiments with four to five mice per group. * or **, *p* < 0.05 or 0.01, respectively, versus the low Th cell dose. (**d**) Blood samples from WT mice transferred with effector CTLs and Th cells or from CD4-deficient mice transferred with effector CTLs alone were collected 6 and 60 days post-transfer, respectively. The samples were processed for tetramer staining along with staining with a panel of biotin-conjugated Abs specific for effector or memory markers, and streptavidin-PE-Texas Red and PE-Cy5. PE-tetramer-positive CD8^+^ T cells in the circles (panels b and e and panels j and m) from WT and Ia^b-/-^ mouse groups were further assessed for expression of memory CTL markers such as CD44, CD62L and IL-7R or inhibitory PD-1 molecule (histogram grey filled overlays), respectively. Irrelevant isotype-matched Abs were used as control (dotted thin lines). Value in each panel represents the percentages of positive staining cells versus the control. One representative of two independent experiments is shown.

As previous reports suggest that T-T cell interactions may have immunoregulatory [1,8,16,19,22,23,25] or immunopotentiation [8,14,25,28] effects, we sought to determine the functional consequences of Th-CTL interactions during the contraction phase on the fate of CTLs. Particularly, we focused our attention on CTL survival and development into functional memory cells. In these experiments, equal number of effector CTLs were adoptively transferred with Th (transitional help) or without Th cells into CD4^+^ T cell-sufficient WT or CD4^+^ T cell-deficient mice, where helped and unhelped CTLs may additionally receive non-specific helper signals from naïve CD4^+^ T cells during the contraction phase (Figure 1a). Since CD4^+^ T cell help delivered during the priming phase was shown to be critical for programming memory CTLs [6,9,10], we also tested this effect in our model using effector CTLs alone or CTLs that received help during *in vitro* priming. Using the tetramer assay, all the mice were monitored for CTL survival and memory CTL expansion after boosting with DCova (Figure 1b). In both WT and CD4^+^ T cell-deficient mice, CTLs which received help during priming demonstrated significantly stronger survival and expansion upon boosting (*P*<0.05), corroborating previous observations [6,7,9,10]. Interestingly, transitionally helped effector CTLs also survived and expanded more efficiently than unhelped CTLs both in WT and CD4^+^ T cell-deficient mice (*P*<0.05). Furthermore, increasing the dose of Th cells from 2x10^6^ to 5x10^6^ cells per mouse resulted in corresponding increase of memory CTL pool in both WT and CD4^+^ T cell-deficient mice (*P*<0.01) (Figure 1c), suggesting the dose of Th cells might directly influence the memory CTL frequencies and memory CTL pool generation.

Since a reduced CTL memory pool was observed in the absence of CD4^+^ T cell help, we compared the phenotypes of helped CTLs in WT mice with unhelped CTLs in Ia^b-/-^ mice at days 6 and 60 following the adoptive effector CTL transfer in an attempt to observe correlations of phenotypic differences with CTL survival rates. On day 6, both helped and unhelped CTLs showed almost equal expression of memory CTL markers CD44 and CD62L although IL-7Rα expression was slightly higher in helped CTLs (18.1%) compared to unhelped CTLs (3.9%) (Figure 1d). On day 60, memory CTLs differentiated from helped CTLs in WT and unhelped CTLs in Ia^b-/-^ mice again showed almost similar expression of the above molecules, indicating that effector CTLs are able to differentiate into memory CTLs in both WT and CD4^+^ T-cell-deficient mice although their survival is compromised without CD4^+^ T-cell help. In addition, helpless memory CTLs in Ia^b-/-^ mice significantly upregulated PD-1 expression (56%) (Figure 1d).

**Figure 2:**
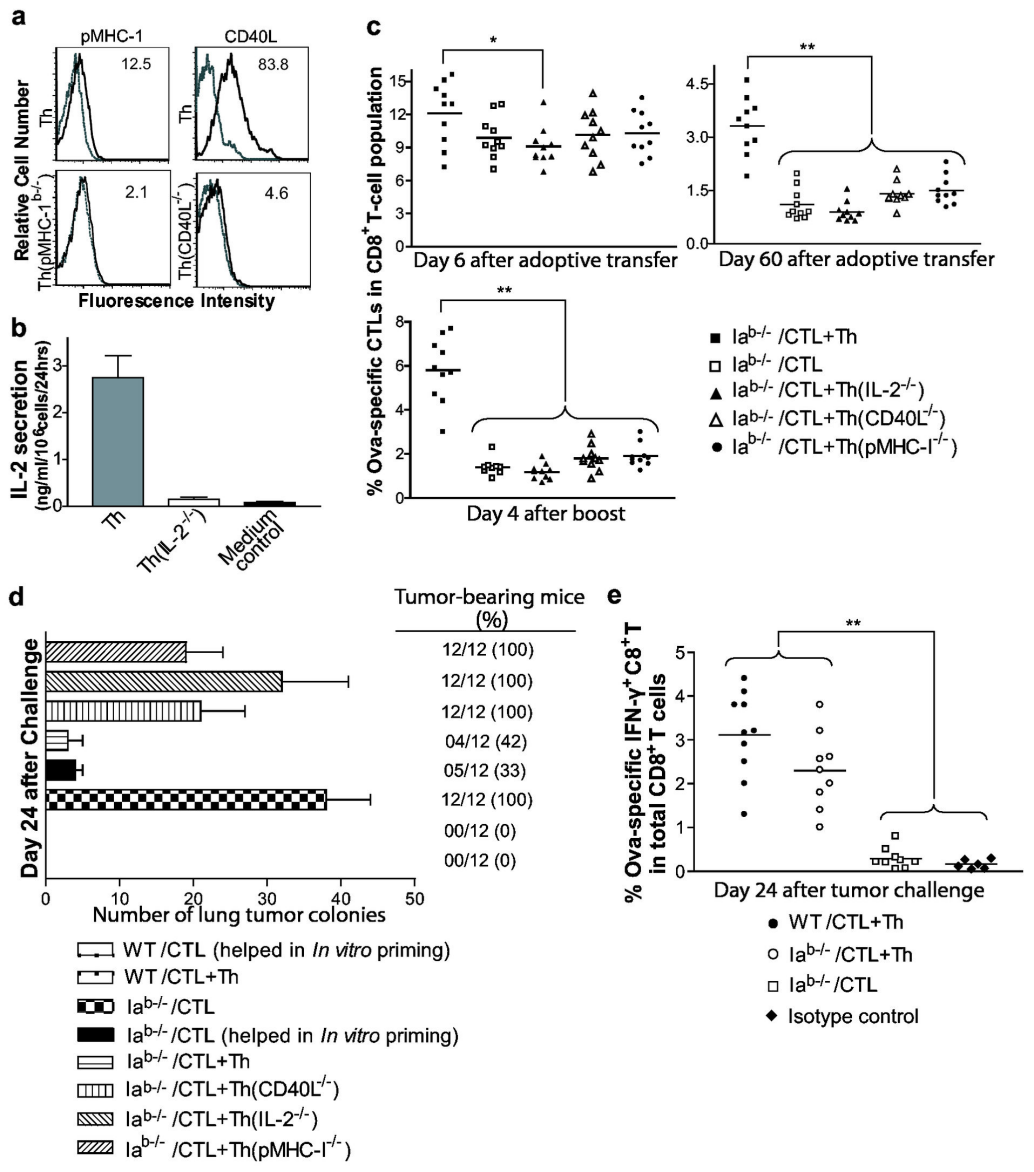
Molecular mechanisms of cognate Th-delivered help responsible for CTL survival and recall responses. (**a**) DCova(K^b–/–^)-stimulated Th(pMHC-I^-/-^) cells were stained with anti-pMHC I Ab and DCova-stimulated Th(CD40L^-/-^) were stained with anti-CD40L Ab (solid thick lines; bottom panels) using DCova-stimulated Th cells as a positive control (solid thick lines; top panel). Stained cells were analyzed by flow cytometry. Irrelevant isotype-matched Abs were used as control (dotted thin lines). Value in each panel represents the percentages of positive staining cells versus the control. One representative of two independent experiments is shown. (**b**) Both Th and Th(IL-2^-/-^) cells were re-stimulated with OVA_323-329_-pusled LB27 cells, and IL-2 secretion was assessed in the culture supernatants. The values represent mean%±SD of IL-2 secreted in supernatant and are cumulative of two independent experiments performed in triplicates. (**c**) Approximately 5x10^6^ effector CTLs were i.v. transferred with or without Th, Th(CD40L^-/-^), Th(IL-2^-/-^) or Th(pMHC I^-/-^) (2x10^6^) cells to CD4^+^ T cell-deficient mice, and analyzed for survival 6 and 60 days later, and recall responses on the 4^th^ day after boosting. The values represent frequencies of tetramer^+^ CTLs in total CD8^+^ T-cell population, and are cumulative of three independent studies with three to four mice per group. The horizontal bars indicate means. * or **, *p* <0.05 or 0.01, respectively, versus Th cell-helped CTLs in Ia^b-/-^ mice. (**d**) Approximately 5x10^6^ effector CTLs alone that received help during priming period or 5x10^6^ effectors CTLs with or without 2x10^6^ Th cells (i.e., help after CTL priming) were i.v. transferred to WT and Ia^b-/-^ mice. In addition, some Ia^b-/-^ mice groups were transferred with effectors CTLs with or without effector Th, Th(IL-2^-/-^), Th(CD40L^-/-^) or Th (pMHC-I^-/-^) cells (2x10^6^each) as indicated. After 100 days, all the mice were challenged i.v. with highly metastasizing BL6-10_OVA_ tumor cells. On 24^th^ day of tumor challenge, the numbers of metastasized tumor colonies in lungs were counted and presented as mean%±(SD). The values represent number and percentage of tumor-bearing mice. The data are cumulative of three independent experiments with three to four mice per group. Note: The data of all PBS-injected control mice, which showed >100 tumor colonies, is not shown. (**e**) Assessment of OVA-specific IFN-γ^+^ CTLs in the spleen of tumor-challenged mice. Approximately 5x10^6^ effector CTLs with Th cells (2x10^6^) or without Th cells were adoptively co-transferred into WT or CD4^+^ T cell-deficient mice. Hundred days later, all the groups were challenged with BL6-10_OVA_ tumor cells. On 24^th^ day of tumor challenge, the spleen samples were analyzed by intracellular IFN-γ^+^ staining to assess CTL-mediated tumor protection. The data represent cumulative frequencies of ova-specific IFN-γ^+^ CTL in total CD8^+^ T cell population. **, *p* < 0.01 versus unhelped CTLs in Ia^b-/-^ mice. One representative of the three independent experiments with three to four mice per group is shown.

As the CD40L and IL-2 molecules provided by Th cells and the Th cell-acquired pMHC-I are known to influence CTL priming [8,31], survival or memory development [2,6–8,32], we attempted to determine whether these Th cell-provided signals have any impact on the fate of effector CTLs. It is becoming increasingly clear from various studies [8,21,22,25] that DC-stimulated T cells acquire non-agonistic bystander pMHC complexes in addition to agonistic ones. Acquisition of DC-derived bystander pMHC-I by Th cells could facilitate their direct interactions with effector CTLs in TCR-Ag specific manner. We found that the transfer of bystander pMHC-I onto Th cells occurred on DCova-activated Th cells, whereas, consistent with the previous results [8,21], DCova(K^b-/-^)-stimulated Th(pMHC-I^-/-^) cells failed to display these molecules (Figure 2a). As expected, in contrast to WT Th cells, Th(CD40L^-/-^) and Th(IL-2^-/-^) cells failed to display CD40L expression or IL-2 secretion (Figure 2a, 2b). Th, Th(CD40L^-/-^), Th(IL-2^-/-^) or Th(pMHC-I^-/-^) cells were co-transferred with effector CTLs into CD4^+^ T cell-deficient mice, and CTLs were monitored for their survival and memory development. During the memory stage, effector CTLs transferred alone or with Th(CD40L^-/-^), Th(IL-2^-/-^) or Th(pMHC-I^-/-^) failed to survive, and expand upon boosting (*P*<0.01 versus Th cell-helped CTLs) (Figure 2c). In contrast, helped memory CTLs expanded up to 2 fold from their basal levels, reaching almost 5.8±1.5% of the total CD8^+^ T cell population (Figure 2c).

To further assess the impact of Th cell-triggered signaling on the functionality of memory CTLs, effector CTLs alone that received help during priming or effectors CTLs with or without Th cells (i.e., help after CTL priming) were transferred WT mice. Similarly, effector CTLs alone that received help during priming period or effectors CTLs with or without effector Th, Th(IL-2^-/-^), Th(CD40L^-/-^) or Th(pMHC-I^-/-^) cells were transferred to Ia^b-/-^ mice as indicated. Hundred days later, all the groups were challenged with highly-metastasizing BL6-10_OVA_ tumor cells. In line with the above recall responses, helped CTLs that received help during or after priming completely protected WT mice against tumor challenge (Figure 2d). Similarly, they were able to protect up to 60-70% of the CD4^+^ T cell-deficient mice, and in mice that developed tumors only fewer lung malignant tumor colonies were observed. In contrast to this, all the CD4^+^ T cell-deficient mice transferred with CTLs alone or CTLs together with Th(pMHC-I^-/-^), Th(CD40L^-/-^) or Th(IL-2^-/-^) cells failed to get protection, and had considerably increased tumor colonies of varying sizes in lungs (Figure 2d). The origin of tumor colonies was further verified by histopathology (data not shown). To further confirm the tumor protection conferred was actually provided by helped CTLs, we tracked OVA-specific IFN-γ^+^ CTLs in spleens at day 24 subsequent to tumor cell challenge by intracellular anti-IFN-γ Ab staining. As expected, IFN-γ^+^ CTLs that received help after priming were present in significantly proportions (*P*<0.01 versus unhelped CTLs; Figure 2e) in both WT and CD4^+^ T cell-deficient mice. Together, these data suggest a possible role for cognate Th cells in the CTL survival and functional memory development.

**Figure 3:**
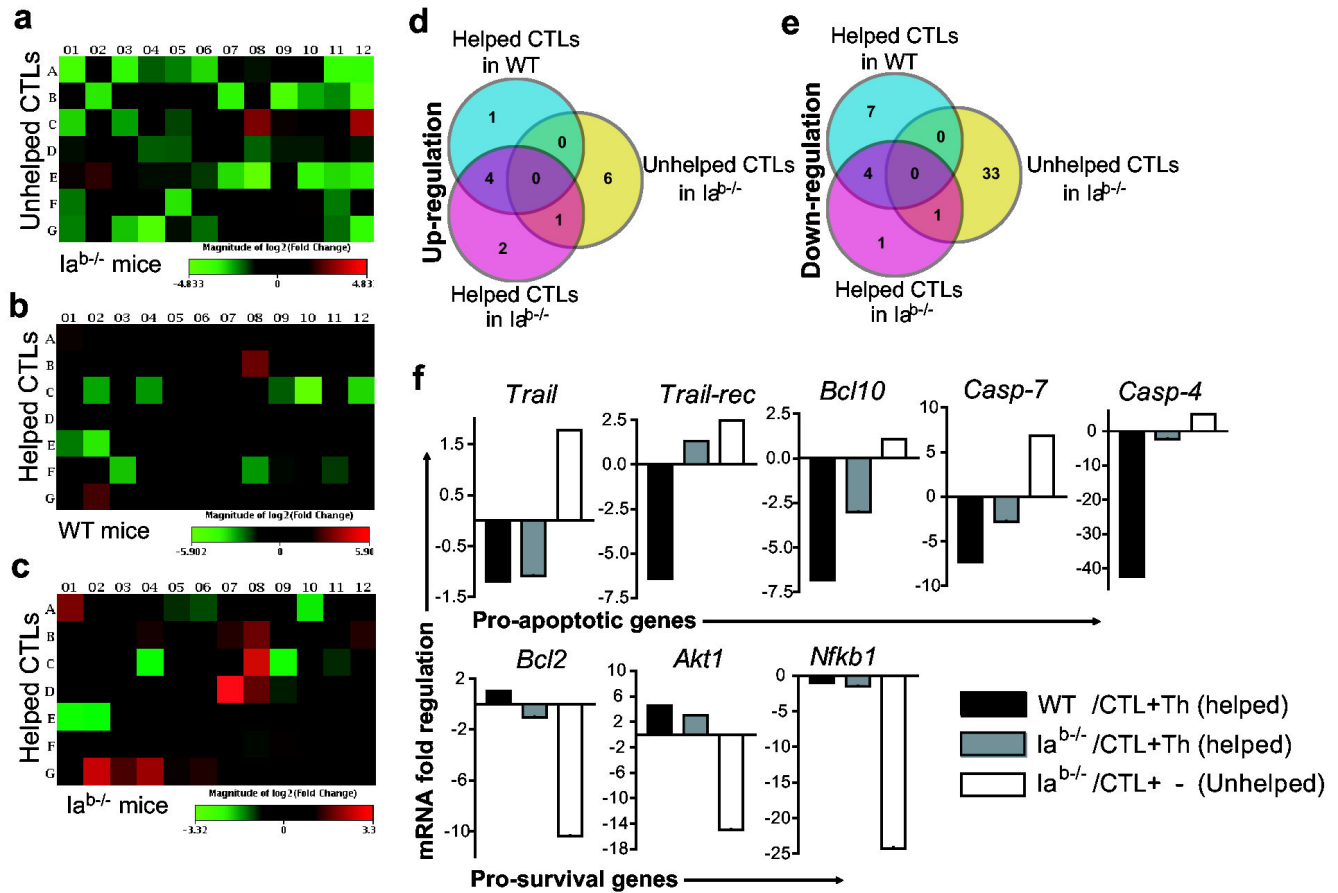
The provision of Th help causes distinct changes in expression of genes that regulate apoptosis, favoring effector CTL survival. (**a**–**c**) Apoptosis pathway-focused gene expression in helped versus unhelped CTLs. Total RNA from purified population of helped or unhelped CTLs was isolated, reverse transcribed to cDNA, and subjected to PCR array. Heat map showing relative gene expression in helped or unhelped CTLs where intensity of color towards red indicates up-regulation and green indicates down-regulation. One representative figure from each group in two independent experiments is shown. Each sample was run on duplicates using pooled cDNA samples derived from two to four mice per group. (**d**–**e**) Statistically significant changes in gene expression (at least three-fold mRNA difference compared to naïve OTI CD8^+^ T cells), up- (elevated-top) or down-regulated (reduced-bottom) and their overlap between helped or unhelped CTLs purified from CD4^+^ T cell-sufficient or CD4^+^ T cell-deficient mice are shown, as indicated. See Tables S2a and S2b for gene-expression data. (**f**) Validation of PCR array results by qRT-PCR. cDNA samples from helped or unhelped CTLs used for PCR array were subjected to qRT-PCR using SYBR Green detection protocol. mRNAs up- or down-regulated in helped or unhelped CTLs purified from CD4^+^ T cell-sufficient or CD4^+^ T cell-deficient mice are shown as indicated.

Although both helped and unhelped CTLs showed a very similar memory marker expression patterns (Figure 1d), helped CTLs survived considerably better. We hypothesized that CD4^+^ T helper cells might trigger distinct molecular mechanisms to influence CTL survival. To gain mechanistic insights into enhanced survival of helped CTLs, we compared the mRNA profiles in three groups of CTLs. These include helped CTLs purified from WT or Ia^b-/-^ mice, and unhelped CTLs purified from Ia^b-/-^ mice. On days 13-18 following the adoptive transfer, we observed a moderate contraction in effector CTL populations. During this period, we purified CTLs from blood and lymphoid organs of WT or CD4^+^ T cell-deficient mice, respectively. Flow cytometric assessment showed 94-96% purity of obtained cells (Figure **S2**). Purified CTLs were subjected to apoptosis pathway-focused PCR arrays, which analyze expression of genes coding for *TNF* family ligands and their receptors, members of the *Bcl*, *Caspases*, *IAP*, *TRAF*, *CARD*, death domain, death effector domain, and *CIDE* families as well as of genes involved in the p53- and ATM-controlled pathways. Interestingly, two distinct patterns of gene expression were observed. First, unhelped CTLs generally showed up-regulation of pro-apoptotic and down-regulation of pro-survival genes (Figure **3a, 3d** **, 3e;** Table **S2a** **, S2b**). Among the up-regulated genes, Caspase family genes, *Casp-7, -2* and *-3*, TNF family members *Tnfrsf1a* and *Trp53*, which mediate apoptosis induction, were prominent. Interestingly, 34 genes were found to be significantly down-regulated, of which 24 were pro-survival and 10 were pro-apoptotic genes. Some of the most prominent pro-survival genes down-regulated include *Akt1, Api5, Bag1, Bcl-2, Birc3 and 5, Nfκb1, Nol3, Pak7, Pim3, Traf1*, and *Zc3hc1*. The down-regulation of some pro-apoptotic genes, although surprising, may represent compensatory mechanisms that occur in cells experiencing various stresses in an attempt to prevent cell death. Second, in contrast to the situation observed in unhelped CTLs, there was a notable shift in the gene-expression profile in helped CTLs, favoring their survival (Figure **3b–3e** Table **S2a** **, S2b**). Interestingly, a considerable overlap was observed in the up- and down-regulated genes in helped CTLs derived from WT and CD4^+^ T cell-deficient mice (Figure 3d, 3e), suggesting a similar role for effector Th cells in both WT and CD4^+^ T cell-deficient mice. Among the up-regulated genes, *Akt1, Xiap, CD40lg* and *Traf1*, which mediate signals involved in apoptosis inhibition, were prominent. Additionally, helped CTLs from Ia^b-/-^ mice exhibited the up-regulation of the anti-apoptotic gene, *Dad1*, and the pro-apoptotic genes, *Casp-2* and *Dapk1*. Among down-regulated genes, the Caspase superfamily genes, *Casp-4, -7* and *-3*, the *TNF* superfamily member *Tnfrsf10b*, and the Caspase recruitment domain superfamily members *Card6*, and *Bcl-10*, all involving in apoptosis induction, were observed.

**Figure 4:**
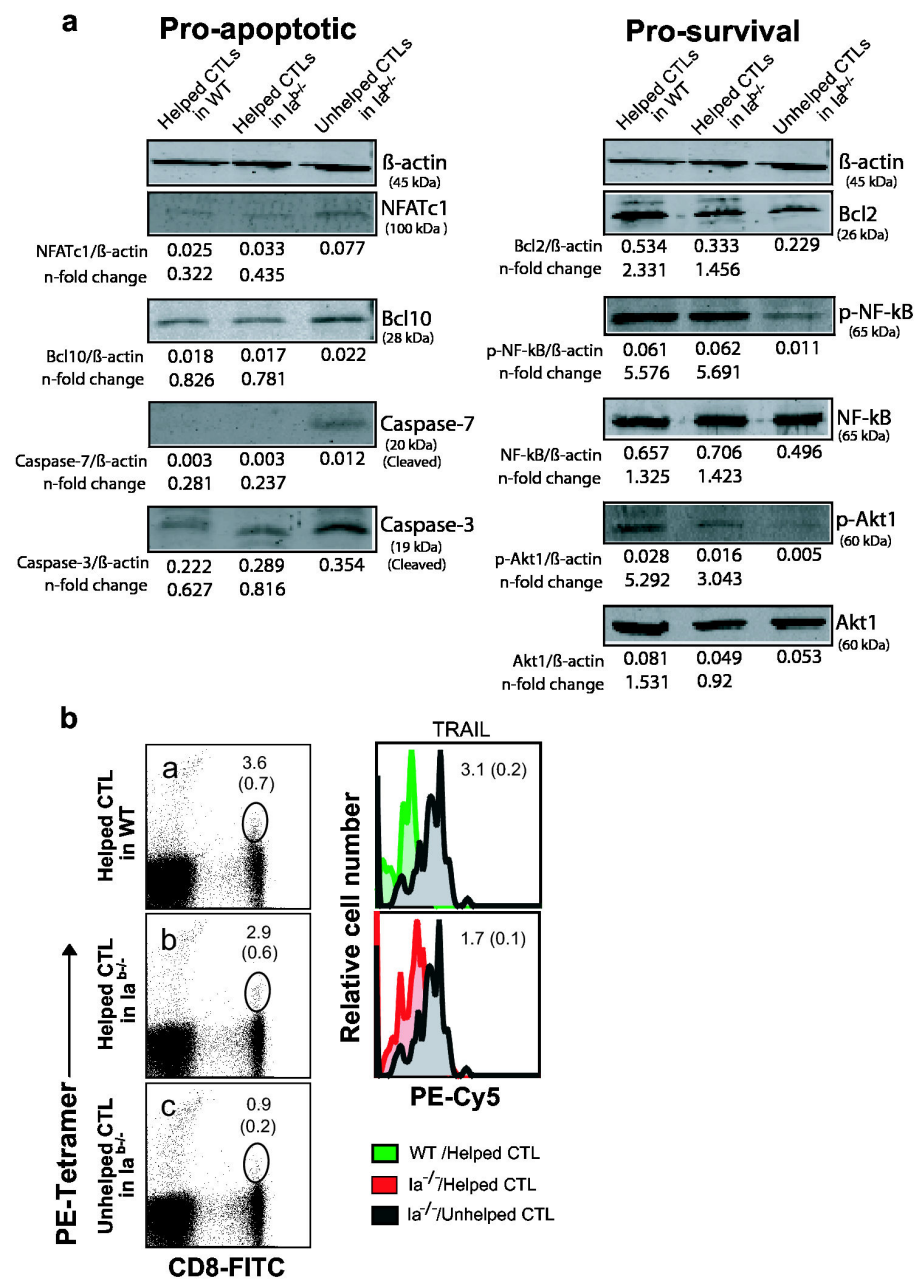
Immunoblot analysis of the expression and phosphorylation status of pro-survival and pro-apoptotic proteins. (**a**) Lysates prepared from helped or unhelped CTLs obtained from WT or CD4^+^ T cell-deficient mice were subjected to SDS-PAGE, and transferred to the nitrocellulose membrane. Western blotting was performed with a panel of Abs specific for β-actin, Bcl-2, Bcl10, Akt1, NF-κB-p65, phosphorylated-Akt1 and -NF-kB-p65, cleaved Caspases-3 and -7, or NFATc1 transcription factor and analyzed by the ODYSSEY densitometer. Densitometric values were normalized on the β-actin control and n-fold changes of normalized targets in the helped CTLs of WT or Ia^b-/-^ mice relative to the unhelped CTLs in Ia^b-/-^ mice are shown below the corresponding lanes. Data are derived from samples pooled from four to six mice in each group of the first experiment. The results of the second experiment are consistent with the first experiment (data not shown). One representative of two independent experiments is shown. (**b**) Representative FACS plots of helped CTLs from WT or CD4-deficient mice, or unhelped CTLs from CD4-deficient mice. Blood samples from helped CTLs in WT and Ia^b-/-^ mice and unhelped CTL in Ia^b-/-^ mice were collected 16 days of adoptive transfer and processed for triple staining. Value in panel a, b and c represents percentage of tetramer^+^-and CD8^+^-specific double-positive cells in the total of CD8^+^ T cell population. The tetramer^+^-and CD8^+^-specific double-positive cell populations were gated (in the circle) for analysis of TRAIL expression. Histogram overlays (right panels) of CD8^+^ tetramer^+^-gated helped CTLs from WT (histogram green filled overlays; right top panel) or CD4-deficient mice (histogram red filled overlays; right bottom panel) and CD8^+^ tetramer^+^-gated unhelped CTLs from CD4-deficient mice (histogram grey filled overlays; right top and bottom panel). The mean of fluorescence intensity (MFI) was calculated from triplicate values. The value in each panel represents the mean of MFI ± SD of positive staining cells (grey) versus the controls with MFI of 0.5 for green and MFI of 0.8 for red, respectively. One representative of two independent experiments is shown.

To further provide key insights into the above gene-expression studies, some of the key genes associated with cell apoptosis or survival, such as *TRAIL, TRAIL-rec, Caspase-4, Caspase-7, Bcl-10, Bcl-2, Akt1* and *Nfκb1*, were further analyzed individually in helped and unhelped CTLs by qRT-PCR using the same cDNA samples. We found that helped CTLs showed up-regulation of the prosurvival (*Bcl-2, Akt1 and Nfκb1*) and down-regulation of the proapoptotic (*TRAIL, TRAIL-rec, Bcl-10, Caspase-4 and Caspase-7*) genes (Figure 3f). Two major pathways have been reported to be associated with cell apoptosis [33]. These include: (i) the intrinsic pathway, regulated by Bcl-2 family members [34] and the extrinsic pathway, triggered by death receptor (TRAIL)-mediated activation of pro-Caspase-8, which subsequently activates effector Caspases [35]. We analyzed various key molecules that regulate these pathways, including NFATc1, Bcl-10, Caspase-3, and -7, Bcl-2, NF-κB-p65, Akt1 and TRAIL as well as the phosphorylated form of NF-κB-p65 and Akt1 involved in cell apoptosis or survival were analyzed in helped and unhelped CTLs by Western blotting and flow cytometry. Consistent with PCR array and qRT-PCR results, we found that helped CTLs displayed an increased expression of Bcl-2, Akt1 and NF-κB as well as the phosphorylated-Akt1 and -NF-κB molecules, while reducing expression of NFATc1, Bcl-10, and cleaved Caspase-3 and -7 molecules in comparison to unhelped CTLs (Figure 4a). Although some discrepancies between the mRNA and protein levels of NF-κB and Akt1 were observed, these most probably result from posttranscriptional and posttranslational modifications that may interfere with the direct mRNA to protein translation [36]. Furthermore, we found that unhelped CTLs in Ia^b-/-^ mice demonstrated increased expression of TRAIL (MFI, 3.1±0.2 or 1.7±0.1) in comparison to helped CTLs in either Ia^b-/-^ or B6 mice (MFI, 0.5 or .8) (Figure 4b). Our data thus indicate that Th cells could rescue effector CTLs from AICD in the contraction phase by inducing up-regulation of the anti-apoptotic genes and down-regulation of the pro-apoptotic genes, that regulate both intrinsic and extrinsic pathways involved in programmed cell death.

## Discussion

Identifying specific factors derived from helper CD4^+^ T cells involved in the regulation of effector CTL responses is currently one of the most active areas in the immunological research [11]. In the last two decades, the role of signaling by CD4^+^ T cells through CD40L and IL-2 molecules in the priming phase for memory CTL development has been extensively studied [4,32,37]. Tanchot and colleagues and our group previously demonstrated that Th cells can directly provide CD40L [6–8] and IL-2 [8] to naïve CD8^+^ CTLs in the priming phase for efficient memory CTL development, whereas de Goer de Herve et al. reported that hetero-specific Th cells can rescue effector CTLs [38], and promote memory CTL recall responses by direct cellular contact, relying on CD40L and IL-2 signals [38]. It has recently been shown that CD4^+^ T cell regulation of CTL’s IL-2R expression control CTL differentiation and survival [39,40], thus indirectly supporting the critical effect of IL-2 on CTL memory. In addition to IL-2, CD4^+^ Th cell’s IL-21 signaling has also been demonstrated to be critical for CD8^+^ T cell survival [41]. In this study, we demonstrate that cognate CD4^+^ T cell help required for effector CTL survival and memory programming in the effector phase is also mediated via direct CD40L- and IL-2-induced signaling. In addition, both helped and unhelped CTLs showed almost equal expression of memory CTL markers although IL-7Rα expression was slightly higher in helped CTLs at day 6 subsequent to T cell transfer. It is possible that a slight increase in the IL-7Rα expression in the effector stage (day 6) might have played some role in subsequent survival of helped CTLs as IL-7Rα is known to influence CTL survival [42,43]. During the memory stage, effector CTLs transferred alone or with Th(CD40L^-/-^), Th(IL-2^-/-^) or Th(pMHC-I^-/-^) failed to survive, and expand upon boosting. In contrast, helped memory CTLs expanded up to only 2 fold from their basal levels, reaching almost 5.8±1.5% of the total CD8^+^ T cell population, reflecting the contribution of endogenous CD4^+^ T cell help at the time of boost [45]. In addition, the persistence of memory CD4^+^ T cells derived from adoptively transferred CD4^+^ Th cells may also rescue functional recall responses. Therefore, the failure of recall responses in mouse groups with transfer of CD4^+^ Th(IL-2^-/-^) and Th(CD40L^-/-^) cells may also be a result of lacking help IL-2 and CD40L signaling [12,13]. In this study, we also demonstrate that unhelped memory CTLs in Ia^b-/-^ mice expressed inhibitory PD-1 molecule, which is consistent with another recent report by Fuse et al, showing that helpless memory CTLs upregulated PD-1 expression and failed in recall responses [44]. These observations provide a strong evidence to support the recently proposed models, suggesting that both quantity and quality of memory CTLs can be altered in the effector phase [12,13]. The work of Kennedy et al. also showed that CD4^+^ Th cells rescued CTL from AICD via CD4^+^ Th cell-to-CD8^+^ T cell contact [12,13]. In addition to prolonged CTL survival, how CD4^+^ Th cell’s CD40L and IL-2 signaling affect CTL phenotype and memory development is still unknown. Conducting experiments to address this question is underway in our laboratory. Furthermore, how CD4^+^ Th cell’s helper signals such as IL-2 and CD40L are delivered to cognate CD8^+^ T cells *in vivo* to govern CTL survival leading to functional memories is also not well described.

We previously demonstrated that CD4^+^ T cells acquired Ag-presenting machinery such as pMHC-I complexes during DC stimulation [8,21,27]. The potential pathways for acquisition of pMHC-I by Th cells include (i) internalization and recycling of synapse composed molecules, (ii) dissociation-associated pathway, and (iii) uptake of DC-released exosomes and Ag peptide [15]. The acquisition of pMHC-I complexes appears to be critical for the ability of Th cells to make efficient contact with CTLs, and to deliver CD40L and IL-2 signaling to these effectors in an Ag-specific manner since, in the absence of pMHC-I, a reduced memory CTL pool exhibiting poor recall functions is observed, indicating the primary role for pMHC-I in targeting CD4^+^ T cell help to cognate CD8^+^ T cells. Our recent two photon imaging study provides a strong *in vivo* evidence, showing that CD4^+^ Th cells with acquired pMHC-I, but not CD4^+^ Th cells without acquired pMHC-I are capable of directly interacting with cognate naïve CD8^+^ T cells *in vivo* [45]. This evidence indicates that CD4^+^ Th cells with acquired pMHC-I can also directly interact with effector CD8^+^ T cells *in vivo* to deliver helper signaling. Therefore, the results of this study strongly support the recent emerging notion of the substantial role of Th cells in rescuing effector CTLs from AICD and in promoting the functional memory generation [2,14,41,46]. Perhaps, even in the classical three-cell interactions, Th cells, after detaching from DCs, might directly act on CTLs in the vicinity due to pMHC-I and TCR avidities. Recently, the role for cognate Th cells, acting through the secretion of IFN-γ and chemokines was proposed in mobilizing differentiated effector CTLs into infected tissues [47]. It is very likely that these factors, together with high-affinity pMHC-I and TCR interactions, greatly favor the ability of Th cells to deliver CD40L and IL-2 signals directly to effector CTLs *in vivo*.

Previous reports [38] together with our current study demonstrate that Th cells appear to mediate CTL survival by secreting survival factors, such as IL-2, and by providing costimulation signals including CD40L. However, the intracellular molecular mechanisms, underlying the Th-mediated CTL survival are not completely understood. Recently, CD4^+^ T cell-provided help was shown to be required for effector CTL survival via the regulation of TRAIL and Bcl-x_L_ [2,9,14]. In this study, we performed a more systematical assessment, based on the gene-expression profiling by PCR array, to provide mechanistic insights into the molecular organization of CD4^+^ T cell help effects on CTL fates. Our data show that the poor survival of unhelped CTLs correlates well with the down-regulation of key survival genes, which mediate signals both at nuclear and cytoplasmic levels, that are required for the activation of the pro-survival NF-κB and attenuation of the pro-apoptotic JNK pathways [48]. In contrast, helped CTLs display an increased activation of Akt1 and NF-κB proteins, inducing transcription of various survival genes [48,49]. Interestingly, increased expression of survival genes was not a unique feature of helped effector CTLs, as their mRNA profiles characterized by pro-survival gene expression patterns were almost identical to those of naïve OTI CD8^+^ T cells. A few genes up-regulated above naïve T cell levels, including *Akt1, Xiap, CD40lg* and *Traf1*, might have played a leading role in the effector CTL survival. The serine–threonine kinase Akt1, for example, executes potent antiapoptotic effects by inhibiting a cohort of cell death-inducing molecules and activating multiple anti-apoptotic factors [50,51]. Akt-1 is shown to activate Inhibitor of NF-κB kinase (IKK), which enables the translocation of NF-κB to the nucleus where it drives the transcription of antiapoptotic genes such as Bcl-2 and Bcl-X_L_ [50,52,53]. More recently, it has also been demonstrated that Th-derived IL-21 triggers the activation of the STAT1 and STAT3 pathways and increases expression of the prosurvival molecules Bcl-2 and Bcl-xL in CD8^+^ T cells, leading to efficient CTL survival [41]. Our data are thus consistent with a previous report, showing that down-regulation of Notch-1 and Jagged-1 induces the cell intrinsic apoptotic pathway via the inactivation of Akt and NF-κB [54]. It was previously shown that memory CD8^+^ T cells lacking CD4^+^ T cell help express TRAIL and die by TRAIL-mediated cell death [9,55]. In this study, we demonstrate that unhelped effector CD8^+^ T cells also express TRAIL, indicating that TRAIL-mediated extrinsic apoptotic pathway appears to be another key mediator in the contraction of adoptively transferred effector CTLs.

Adoptive CD8^+^ T cell therapy refers to an immunotherapy approach in which tumor-infiltrating lymphocytes (TILs) isolated from tumor specimen are activated and expanded *in vitro* to large quantities, using anti-CD3/CD28 antibodies and IL-2, and then transferred back to the cancer patients [56]. Alternatively, CTL clones derived from TILs or T cell clones engineered to express recombinant TCR specific for tumor Ag could be used for the adoptive T cell therapy [57–61]. To date, the adoptive CTL cancer immunotherapy, relying on *in vitro-*expanded tumor-infiltrating CD8^+^ T cells, has achieved some degree of success [62,63]. However, one of the major obstacles in this therapy is the poor survival of transferred TILs or CTL clones, and the development of corrupted CTL memories [62,63] due to AICD and the CD4^+^ T cell-deficient environment, typically associated with primary cancer therapies [18,33]. It has been demonstrated that effector CD4^+^ T cells promoted CTL survival and tumor localization via rendering tumor environment permissive in the course of adoptive CTL therapy [2,64]. Therefore, the poor survival of unhelped CTLs associated incomplete protection against lethal tumor challenge shown in this study re-emphasizes the importance of either using Th cells in TIL preparation or co-transferring TILs with Th cells into cancer patients during the adoptive CTL therapy.

Taken together, our results reveal a previously unexplored mechanism of Th cell action in programming CTL responses. The observed Th cell-effector CTL cooperation could explain why memory CTLs, generated under cognate Th cell help, obtain significant survival and recall advantages. Moreover, this knowledge could aid in the development of efficient adoptive CTL cancer therapeutics.

## Supporting Information

Figure S1 Phenotypic characterization of DCova, Th cells and effector CTLs. a. DCova, naïve and DCova-stimulated CD4^+^ (effector Th) and CD8^+^ (effector CTL) T cells (solid thick lines) were stained with a panel of Abs specific for acquired pMHC I and markers for T cell subsets, naïve, activation/maturation or memory T cells as indicated. The cells were then analyzed by flow cytometry. One representative of the two independent experiments is shown. b. DCova and Th cells or effector CTLs were stained with PE-anti-CD11c and FITC-anti-CD4 or FITC-anti-CD8 Abs. The value in the panel represents the mean%±(SD) of CD4^high^CD11c^low^ (left) or CD8^high^CD11c^low^ (right) cells in the total population of CD4^high^ Th or CD8^high^ effector CTLs, respectively. One representative figure of three independent experiments is shown.

Figure S2 Purity of tetramer-enriched CTLs. Approximately 5x10^6^ effector CTLs with Th cells (2x10^6^) or without Th cells were adoptively co-transferred to CD4^+^ T cell-sufficient or CD4^+^ T cell-deficient mice. On the 16^th^ day, the transferred helped or unhelped effector CTLs were purified from blood and lymphoid organs of CD4^+^ T cell-sufficient or CD4^+^ T cell-deficient mice by positive selection using PE-H-2K^b^/OVA_257-264_ tetramer and anti-PE microbeads. The purified CTLs were stained with FITC-anti-CD8 Ab and analyzed for purity by flow cytometry. The data represent mean% ±(S.D) and are cumulative of three independent experiments with two to six mice per group.

Table S1 Related to Figure 5.

Table S2 Related to Figure 5. A. Top genes uniquely up-regulated above 3 fold. B. Top genes uniquely down-regulated below 3 fold.
