# Supplementary material for: Th Cells Promote CTL Survival and Memory via Acquired pMHC-I and Endogenous IL-2 and CD40L Signaling and by Modulating Apoptosis-Controlling Pathways
Source: PLoS One. 2013 Jun 13;8(6):e64787. doi: 10.1371/journal.pone.0064787 (PMC3681805; doi:10.1371/journal.pone.0064787)
Supplement: Table S1 [file pone.0064787.s003.doc]

**Table S1, related to Figure 5**

| Primers | Tm | Sequence (5’ – 3’) | GeneBank ID |
| --- | --- | --- | --- |
| *Akt1* | 58.0 | F - CTTCTATGGTGCGGAGATTGTG | NM_009652 |
|  | 59.0 | R - CCCGGTACACCACGTTCTTC |  |
| *Bcl-10* | 59.0 | F - GAAAGCTGCCGACACACTCA | NM_009740 |
|  | 59.0 | R - CCCGACGGCTTCTCAGAAC |  |
| *Casp-4* | 60.0 | F - CAATGGCCGTACACGAAAGG | NM_007609 |
|  | 58.0 | R - GCCCCATACCTCAGTGAGAGAT |  |
| *Casp-7* | 59.0 | F - CCACCAGCGCCTTATAATTCC | NM_007611 |
|  | 58.0 | R - ATGGTCCCTAGGCCCTCACT |  |
| *Nfkb1* | 59.0 | F - CCAGCTTCCGTGTTTGTTCAG | NM_008689 |
|  | 60.0 | R - TCAGGGTAGTAGAGAAAGGGTTTCG |  |
| *Trail-rec (Tnfrsf10b)* | 59.0 | F - GGGCCTCACAGACAATCAAATC | NM_020275 |
|  | 60.0 | R - GCCTCACGTGTGACCAGTGT |  |
