# Supplementary material for: Th Cells Promote CTL Survival and Memory via Acquired pMHC-I and Endogenous IL-2 and CD40L Signaling and by Modulating Apoptosis-Controlling Pathways
Source: PLoS One. 2013 Jun 13;8(6):e64787. doi: 10.1371/journal.pone.0064787 (PMC3681805; doi:10.1371/journal.pone.0064787)
Supplement: Table S2 — A. Top genes uniquely up-regulated above 3 fold. B. Top genes uniquely down-regulated below 3 fold. [file pone.0064787.s004.doc]

**Table S2A, related to Figure 5.** Top genes uniquely up-regulated above 3 fold

| **Gene Symbol** | **Fold Regulation** | ***p*-value** | **Key functions** |
| --- | --- | --- | --- |
| **Helped CTL in CD4-sufficient mice** | | | |
| *Akt1* | 4.212 | 0.0044 | Inhibit apoptosis via phosphorylation of components of apoptosis pathway |
| *Xiap* | 9.412 | 0.0011 | Inhibit apoptosis via inhibition of caspase 3, 7 and 9 |
| *Prdx2* | 3.462 | 0.0114 | Antioxidant protective role in cells |
| *Cd40lg* | 6.537 | 0.0094 | Expressed on activated T cells, exerts diverse effects depending on type of cells involved |
| *Traf1* | 3.838 | 0.0096 | Activates MAPK8/JNK and NF-kB and mediates anti-apoptotic signals by inhibiting inhibitor-of-apoptosis proteins |
| **Helped CTL in CD4-deficient mice** | | | |
| *Akt1* | 3.524 | 0.0153 | Inhibit apoptosis via phosphorylation of components of apoptosis pathway |
| *Xiap* | 3.164 | 0.0087 | Inhibit apoptosis via inhibition of caspase 3, 7 and 9 |
| *Casp2* | 6.674 | 0.0042 | Induce apoptosis by associating with several pro-apoptotic proteins |
| *Dad1* | 9.983 | 0.0021 | Inhibits apoptosis possibly by interacting with Mcl-1 (a bcl-2 family member) ^1^ |
| *Dapk1* | 3.013 | 0.0199 | Positive mediator of IFN-γ-induced programmed cell death |
| *Cd40lg* | 6.215 | 0.0312 | Expressed on active T cells, exerts diverse effects depending on type of cells involved |
| *Traf1* | 4.424 | 0.0186 | Activates MAPK8/JNK and NF-kB and mediates anti-apoptotic signals by inhibiting inhibitor-of-apoptosis proteins |
| **Unhelped CTL in CD4-deficient mice** | | | |
| *Casp2* | 7.114 | 0.0106 | Induce apoptosis by associating with several pro-apoptotic proteins |
| *Casp3* | 3.246 | 0.0226 | Mediates apoptosis in both extrinsic (death ligand) and intrinsic (mitochondrial) pathways ^2^ |
| *Casp7* | 9.764 | 0.0162 | Executioner protein of apoptosis |
| *Fas (CD95)* | 3.275 | 0.0044 | Induces apoptosis on binding by [Fas ligand](http://en.wikipedia.org/wiki/Fas_ligand). |
| *FasL (CD95L)* | 4.056 | 0.0371 | Induce apoptosis by binding to Fas receptor (Regulate immune system via inducing apoptosis) |
| *Tnfrsf1a* | 3.193 | 0.0332 | Activate [transcription factor](http://en.wikipedia.org/wiki/Transcription_factor) [NF-κB](http://en.wikipedia.org/wiki/NF-κB), mediate [apoptosis](http://en.wikipedia.org/wiki/Apoptosis), and function as a regulator of [inflammation](http://en.wikipedia.org/wiki/Inflammation). |
| *Trp53 (p53)* | 3.014 | 0.0143 | Mediate apoptosis following activation by myriad of stressors |

**Table S2B, related to Figure 5.** Top genes uniquely down-regulated below 3 fold

| **Gene Symbol** | **Fold Regulation** | ***P*-value** | **Key functions** |
| --- | --- | --- | --- |
| **Helped CTL in CD4-sufficient mice** | | | |
| *Bcl10* | -14.122 | 0.0025 | Interact with CARD domain containing proteins including CARD9, 10, 11 and 14, and mediate apoptosis via NF-kB activation^3^ |
| *Card10* | -8.342 | 0.0051 | Interact with Bcl10 and activate NF-kB to induce apoptosis^3^ |
| *Card6* | -7.297 | 0.0021 | Interacts with Cardiac and Nod1 and specifically impairs their ability to induce the transcription factor NF-κB^4^ |
| *Casp3* | -5.025 | 0.0017 | Mediates both extrinsic (death ligand) and intrinsic (mitochondrial) apoptotic pathways^2^ |
| *Casp4* | -59.804 | 0.0002 | Inflammatory caspase, implicated in endoplasmic-reticulum stress-induced apoptosis |
| *Casp7* | -11.942 | 0.0002 | Executioner protein of apoptosis |
| *Fas* | -5.964 | 0.0057 | Induces apoptosis upon binding by [Fas ligand](http://en.wikipedia.org/wiki/Fas_ligand)^5^ |
| *FasL* | -13.781 | 0.0051 | Induce apoptosis by binding to Fas receptor (Immune regulation)^5^ |
| *Pycard* | -10.111 | 0.0022 | Involved in inflammatory and cell death pathways in concert with Caspase-1 |
| *Tnfrsf10b (Trail-rec)* | -7.399 | 0.0056 | Mediate apoptosis following activation by TRAIL^6^ |
| *Tnfrsf11b* | -3.017 | 0.0231 | Exhibit paracrine survival functions on cells^7^ |
| **Helped CTL in CD4-deficient mice** | | | |
| *Bcl10* | -3.059 | 0.0335 | Interact with CARD domain containing proteins including CARD9, 10, 11 and 14, and mediate apoptosis via NF-kappaB activation^3^ |
| *Birc5* | -3.01 | 0.0052 | Prevent apoptosis by inhibiting Caspase activation |
| *Card6* | -3.708 | 0.0012 | Interacts with Cardiak and Nod1 and specifically impairs their ability to induce the transcription factor NF-κB^4^ |
| *Casp3* | -5.231 | 0.0015 | Mediates apoptosis in both extrinsic (death ligand) and intrinsic (mitochondrial) pathways^2^ |
| *Fas* | -3.914 | 0.0091 | Induces apoptosis on binding by [Fas ligand](http://en.wikipedia.org/wiki/Fas_ligand)^5^ |
| *FasL* | -3.681 | 0.0019 | Induce apoptosis by binding to Fas receptor (Regulate immune system)^5^ |
| **Unhelped CTL in CD4-deficient mice** | | | |
| *Akt1* | -14.993 | 0.0191 | Inhibit apoptosis via phosphorylation of components of apoptotic pathway^8,9^ |
| *Api5* | -9.923 | 0.0005 | Promote cell survival by inhibiting apoptosis |
| *Atf5* | -3.695 | 0.0384 | Promote cell survival by inhibiting apoptosis |
| *Bad* | -4.491 | 0.0012 | Involved in initiation of apoptosis |
| *Bag1* | -7.675 | 0.0127 | Enhances the anti-apoptotic effects of BCL2 and represents a link between growth factor receptors and anti-apoptotic mechanisms. |
| *Bcl2* | -11.071 | 0.0013 | Prevent apoptosis^10^ |
| *Bcl2l1* | -10.706 | 0.0018 | Prevent apoptosis by controlling the production of reactive oxygen species and release of cytochrome C by mitochondria |
| *Bcl2l2* | -8.651 | 0.0118 | Prevent apoptosis |
| *Birc3* | -9.627 | 0.0011 | Prevent apoptosis by interfering with caspases activation |
| *Birc5* | -17.175 | 0.0014 | Prevent apoptosis by inhibiting Caspase activation |
| *Bnip2* | -5.851 | 0.0058 | Prevents apoptosis |
| *Bnip3* | -4.653 | 0.0042 | Exhibits pro-apoptotic functions |
| *Bnip3l* | -19.677 | 0.0006 | Promotes cell-death |
| *Bok* | -7.359 | 0.0016 | Pro-apoptotic molecule involved in regulation of cell cycle |
| *Nod1* | -5.493 | 0.0131 | Involved in triggering innate immune response that drives development of adaptive immune responses |
| *Casp1* | -3.262 | 0.0093 | Inflammatory caspsae involved in triggering apoptosis owing to pro-inflammatory cytokines |
| *Cidea* | -3.747 | 0.0505 | Involved in activation of apoptosis |
| *Cideb* | -3.602 | 0.0097 | Involved in activation of apoptosis |
| *Dapk1* | -3.833 | 0.0055 | Positively mediate IFN-γ induced programmed cell death |
| *Ltbr* | -3.132 | 0.0159 | Mediate apoptosis; involved in development and organization of [lymphoid tissue](http://en.wikipedia.org/wiki/Lymphatic_system#Lymphoid_tissue) and transformed cells |
| *Mcl1* | -7.914 | 0.0020 | A critical anti-apoptotic factor for the survival of T cells at multiple stages *in vivo* |
| *Nfkb1* | -28.496 | 0.0029 | Anti-apoptotic to T cells. MKP-1 is a NF-kappaB-mediated prosurvival effector in attenuating JNK-mediated pro-apoptotic response^8,9^ |
| *Nol3* | -9.897 | 0.0198 | Prevents apoptosis |
| *Pak7* | -7.727 | 0.0029 | Regulation of cytoskeletal dynamics, proliferation, and cell survival signalings |
| *Pim2* | -9.293 | 0.0076 | Promote the growth and survival of nontransformed hematopoietic cells |
| *Polb* | -4.186 | 0.0085 | Performs [base excision repair](http://en.wikipedia.org/wiki/Base_excision_repair) (BER) required for [DNA](http://en.wikipedia.org/wiki/DNA) maintenance, [replication](http://en.wikipedia.org/wiki/DNA_replication), [recombination](http://en.wikipedia.org/wiki/Genetic_recombination), and drug resistance (anti-apoptosis) |
| *Rnf7* | -8.467 | 0.0012 | Anti-apoptosis (antioxidant) |
| *Cd40* | -4.395 | 0.0235 | Immunomodulation - exhibit diverse functions |
| *Tnfsf12* | -4.526 | 0.0049 | Known to mediate both apoptosis induction and suppression |
| *Cd70* | -6.649 | 0.0018 | CD27/CD70 interactions at the T-cell/DC interface prime CD8(+) T cells to become tumor-eradicating cytolytic effectors and memory cells |
| *Traf1* | -18.465 | 0.0015 | Activates MAPK8/JNK and NF-kB and mediates anti-apoptotic signals by inhibiting inhibitor-of-apoptosis proteins^8^ |
| *Traf3* | -4.016 | 0.0069 | TRAF3 potently suppresses canonical (p50-dependent) NF-kB activation and gene expression in vitro and in vivo (anti-apoptosis)^8^ |
| *Trp73 (p53)* | -3.988 | 0.0022 | Induce apoptosis in mammalian cells^11^ |
| *Zc3hc1* | -10.101 | 0.0336 | Anti-apoptotic role in NPM-ALK-mediated signaling events |

**Supplementary references:**

1. Makishima T, Yoshimi M, Komiyama S, Hara N, Nishimoto T. A subunit of the mammalian oligosaccharyltransferase, DAD1, interacts with Mcl-1, one of the bcl-2 protein family. J Biochem. 2000;128:399-405.

2. Salvesen GS. Caspases: opening the boxes and interpreting the arrows. Cell Death Differ. 2002;9:3-5.

3. Wang L, Guo Y, Huang WJ, et al. Card10 is a novel caspase recruitment domain/membrane-associated guanylate kinase family member that interacts with BCL10 and activates NF-kappa B. J Biol Chem. 2001;276:21405-21409.

4. Stehlik C, Hayashi H, Pio F, Godzik A, Reed JC. CARD6 is a modulator of NF-kappa B activation by Nod1- and Cardiak-mediated pathways. J Biol Chem. 2003;278:31941-31949.

5. Kennedy R, Celis E. T helper lymphocytes rescue CTL from activation-induced cell death. J Immunol. 2006;177:2862-2872.

6. Janssen EM, Droin NM, Lemmens EE, et al. CD4+ T-cell help controls CD8+ T-cell memory via TRAIL-mediated activation-induced cell death. Nature. 2005;434:88-93.

7. Shipman CM, Croucher PI. Osteoprotegerin is a soluble decoy receptor for tumor necrosis factor-related apoptosis-inducing ligand/Apo2 ligand and can function as a paracrine survival factor for human myeloma cells. Cancer Res. 2003;63:912-916.

8. Kim EY, Teh SJ, Yang J, Chow MT, Teh HS. TNFR2-deficient memory CD8 T cells provide superior protection against tumor cell growth. J Immunol. 2009;183:6051-6057.

9. Jones RG, Saibil SD, Pun JM, et al. NF-kappaB couples protein kinase B/Akt signaling to distinct survival pathways and the regulation of lymphocyte homeostasis in vivo. J Immunol. 2005;175:3790-3799.

10. Rosse T, Olivier R, Monney L, et al. Bcl-2 prolongs cell survival after Bax-induced release of cytochrome c. Nature. 1998;391:496-499.

11. Martel V, Filhol O, Colas P, Cochet C. p53-dependent inhibition of mammalian cell survival by a genetically selected peptide aptamer that targets the regulatory subunit of protein kinase CK2. Oncogene. 2006;25:7343-7353.
